# Supplementary material for: Evaluating digital literacy of health professionals in Ethiopian health sectors: A systematic review and meta-analysis
Source: PLoS One. 2024 May 16;19(5):e0300344. doi: 10.1371/journal.pone.0300344 (PMC11098478; doi:10.1371/journal.pone.0300344)
Supplement: Attachment — Submitted filename: Reviewer Attachments.docx [file pone.0300344.s005.docx]

1. The importance of the topic is not clearly stated. The author should further describe the current situation of digital literacy of local medical staff and the significance of the topic, with emphasis on the innovation of the topic.

2. The inclusion and exclusion criteria are not clear enough and need to be described in detail. The author should make a more specific elaboration from the types of studies, research methods and outcome indicators.

3. The number of selected papers is relatively small, which will have a certain impact on the accuracy of the results, and the author should explain the corresponding reasons and shortcomings.
