# Supplementary material for: Evaluating digital literacy of health professionals in Ethiopian health sectors: A systematic review and meta-analysis
Source: PLoS One. 2024 May 16;19(5):e0300344. doi: 10.1371/journal.pone.0300344 (PMC11098478; doi:10.1371/journal.pone.0300344)
Supplement: Attachment — Submitted filename: point_by_point.docx [file pone.0300344.s006.docx]

**Dear Editors of PLOS ONE;**

It has been recalled that we the authors of the manuscript entitled **“Assessing Health Professional’s Digital Literacy Level and Associated Factors in Ethiopian Health Sectors: A Systematic Review and Meta-analysis”** submitted our manuscript for publication in your journal received reviewer comments for the betterment of the manuscript before its publication.

In line with this, all authors are very happy with the constructive and valuable comments given by reviewers. Accordingly, we have considered all the comments and provided a point-by-point response and explanations for all the questions raised.

Finally, we have submitted all the required documents in their revised form. We hope that we have addressed all the questions and if you have any points for further clarity, let us know.

All the authors would like to thank you the editorial team and the reviewers.

**Editor(s)' Comments to Authors**

**Answer for reviewer 1**

**Comment 1:** The importance of the topic is not clearly stated. The author should further describe the current situation of digital literacy of local medical staff and the significance of the topic, with emphasis on the innovation of the topic.

**Answer**: Thank you very much for your interesting and supportive comments; we have explained the importance of the topic in detail (pages 3-5).

**Comment 2**: The inclusion and exclusion criteria are not clear enough and need to be described in detail. The author should make a more specific elaboration on the types of studies, research methods, and outcome indicators.

**Answer**: Thank you so much for your constructive comments. We have tried to clarify the comments that you raised to us (page 6, lines 12- 21).

**Comment 3**: The number of selected papers is relatively small, which will have a certain impact on the accuracy of the results, and the author should explain the corresponding reasons and shortcomings.

**Answer**: Thank you for your valuable feedback regarding the number of articles included in our assessment of digital literacy levels. We appreciate your concern regarding the relatively small number of selected papers in our study. However, our extensive search across various databases, browsers, and search engines, we found limited research conducted on the digital literacy level in Ethiopia. This scarcity of available studies is a reflection of the current state of research in this specific context. We acknowledge this limitation and will provide a clear explanation of the reasons and shortcomings in our manuscript. Due to this, we conclude the number of research related to digital literacy levels is limited in Ethiopia. Thank you for bringing this to our attention.

**Answer for reviewer 2**

**Comment 1**: It is suggested to delete the first paragraph of the background as it is not related to the purpose of the study and contains repetitive and old material.

**Answer**: Thank you for your valuable feedback. We have removed the recommended paragraph and briefly explained it in other ways (page 3).

**Comment 2**: In the third paragraph, the text discusses the digital literacy assessment tool. It is unclear why this tool is being discussed and whether its development was one of the items of interest in the study. It would be more effective to focus on digital literacy and its components.

**Answer**: Thank you so much for your supportive comments. We have modified the ideas of the sentence based on the given comment and explained more about digital literacy and digital literacy level (pages 3-4).

**Comment 3**: The last sentences of the last paragraph pertain to the conclusion section.

**Answer**: Thank you for your valuable feedback and detailed comments: we have modified the sentences you suggested to remove (page 5)

**Comment 4**: Explain how to score according to NOS and the number of questions.

**Answer**: Thank you so much for your interesting comment. We have clearly explained and attached the quality assessment criteria questions as supportive material (page 7).

**Comment 5**: The databases reported in this section differ from those mentioned in the method.
The search process appears to have been conducted incorrectly. If Ethiopia is one of the key search terms, as mentioned in the method, studies outside of this country should not have been included in the initial search. It is unclear why studies outside of Ethiopia were excluded in the eligibility stage. To ensure clarity, it is recommended to present the dimensions of digital literacy mentioned in each study in a table format.

**Answer**: Thank you very much for your advisable comment. We have corrected this in the method section. I.e. we have removed the keyword Ethiopia from the method section and modified other keywords (page 6).

**Comment 6**: The study's results should be compared to those of similar studies, and conclusions should be drawn based on the comparison. It is not advisable to repeat sentence A.

**Answer**: Thank you for your valuable feedback. We appreciate your suggestion to compare our study's results to those of similar studies and draw conclusions based on the comparison. However, despite our comprehensive search efforts, we were unable to find any systematic review papers specifically related to digital literacy level. We acknowledge the limitation and understand the importance of comparative analysis. We will explore alternative approaches to effectively compare results and draw meaningful conclusions. Thank you for your guidance in this matter.
