# Supplementary material for: Evaluating digital literacy of health professionals in Ethiopian health sectors: A systematic review and meta-analysis
Source: PLoS One. 2024 May 16;19(5):e0300344. doi: 10.1371/journal.pone.0300344 (PMC11098478; doi:10.1371/journal.pone.0300344)
Supplement: Attachment — Submitted filename: Response to Reviewers.docx [file pone.0300344.s007.docx]

**Dear Reviewers**

On behalf of the authors, I extend our sincere gratitude for your meticulous review of our manuscript, "***Evaluating Digital Literacy of Health Professionals in Ethiopian Health Sectors: A Systematic Review and Meta-Analysis***." Your expertise and insightful feedback have played an instrumental role in enhancing the clarity and quality of our work. Your commitment to excellence and professionalism throughout the review process has not gone unnoticed, and we are deeply appreciative of your dedication. Your willingness to share your expertise has been invaluable to us, and we recognize the significant contribution you have made to our research. Your thorough examination and constructive critique have undoubtedly strengthened the manuscript.

Once again, we express our heartfelt thanks for your invaluable contribution to our work. Your input has been crucial in shaping the final outcome, and we are genuinely grateful for your commitment to advancing scientific knowledge.

**Reviewers' Comments to Authors**

**Answer for reviewers comments**

**Reviewer 2:** The authors have made significant edits, but the discussion section requires further improvement. It should include more references and employ more reasoning and deduction.

**Answer:** Thank you for your valuable feedback and detailed comments regarding discussion section. We have tried to improve based on the given comments (Page 9 line 17 to 20).

Reviewer 3:

**Comment 1:** - It's better to seek help from a native English speaker to edit the text.

**Answer:** Thank you for your valuable feedback on our manuscript, your insights have been incredibly helpful in improving the clarity and quality of our work. We have taken your suggestions seriously, particularly regarding the need for language editing to ensure clear and accurate communication.

To address this, we are currently seeking assistance from a native English speaker to edit the text thoroughly. We appreciate your patience and understanding as we strive to enhance the readability and coherence of the manuscript. Your continued support is invaluable to us, and we are committed to addressing all concerns raised during the review process.

**Comment 2:** The short title is not short enough; it's better to make it shorter.

**Answer:** Thank you for your valuable feedback regarding the short title of our manuscript. We understand the importance of brevity and have made further adjustments to ensure the title is concise and accurately reflects the content of our research. We appreciate your guidance and attention to detail throughout the editorial process.

**Comment 3:** In the background section, remove the letter S at the beginning of the first line.

**Answer**: Thank you for your valuable feedback to removing unnecessary character in our manuscript. We remove the later s in the background section (page 3 line 2)

**Comment 4:** In the background section, the reference numbers should start from 1, but they have started from 11. Please review and edit all the references in manuscript.

**Answer**: Thank you for bringing to our attention the issue with the reference numbers in the background section of our manuscript. We have carefully reviewed and corrected all references to ensure they start from 1 rather than 11. Additionally, we have thoroughly updated the entire manuscript to reflect these changes. We appreciate your diligence in ensuring the accuracy of our work.

**Comment 5:** In the last line of the "eligibility criteria" section, you wrote "Additionally, researchers without editorial reports, letters, reviews, or commentaries were excluded from the study. Why? Please explain about the reason.

**Answer:** Thank you for your insightful feedback regarding the "eligibility criteria." Excluding papers such as non-editorial reports, letters, reviews, or commentaries from the study serves to uphold the integrity and focus of our research. These types of publications typically lack original research data or offer substantive insights into the digital literacy levels of health professionals in Ethiopian health sectors. By omitting them, our study ensures that only primary research studies containing relevant data are included, thereby enhancing the reliability and validity of the systematic review and meta-analysis. This focused approach enables us to gather robust evidence directly contributing to our understanding of digital literacy among health professionals in Ethiopia, rather than including potentially tangential or less relevant information (page 5 lines 21 to page 6 lines 2).

**Comment 6:** In the "eligibility criteria" section, it is stated that studies from 2015 onwards have been considered. Please explain why studies before 2015 were not considered.

**Answer:** Thank you for your insightful feedback regarding the "eligibility criteria." In Ethiopia, the landscape of healthcare and technology has undergone significant transformations in recent years. Before 2015, there were limited initiatives and investments in digital health infrastructure and policies. Consequently, studies conducted before this period may not accurately reflect the current state of digital literacy among health professionals or the effectiveness of related interventions. By focusing on studies from 2015 onwards, we ensure relevance to the contemporary Ethiopian healthcare context, where digital literacy initiatives have gained momentum and are more representative of the current situation. This approach allows us to provide up-to-date and pertinent insights for informing future policies and interventions in the Ethiopian health sector.

**Comment 7:** - The title of a table 1 is written as: Quality evaluation assessment of the selected study. It is better to remove one of the words; “assessment or evaluation”. Evaluation assessment is not correct. Also replace the “study” with “studies”.

**Answer**: Thank you for your valuable feedback to removing unnecessary words in the table caption. We have removed and correct it based on your suggestion (page 6 line 11)

**Comment 8:** Please modify the title of S1 Table 1. Using the words “assessment “and” evaluation " consecutively is not correct.

**Answer**: Thank you for your valuable feedback to removing unnecessary words in the table caption. We have removed and correct it based on your suggestion (page 11 line 27)
